# Low positive and borderline negative transglutaminase antibody levels are frequently associated with a coeliac disease diagnosis

**DOI:** 10.1111/joim.70025

**Published:** 2025-09-25

**Authors:** Rakel Nurmi, Celina Turunen Beteta, Kalle Kurppa, Heini Huhtala, Katri Lindfors, Laura Kivelä, Katri Kaukinen, Saana Paavola

**Affiliations:** ^1^ Celiac Disease Research Center Tampere University Tampere Finland; ^2^ Department of Internal Medicine Tampere University Hospital Wellbeing Services County of Pirkanmaa Tampere Finland; ^3^ Tampere Center for Child Adolescent and Maternal Health Research Tampere University Tampere Finland; ^4^ Department of Pediatrics Tampere University Hospital Wellbeing Services County of Pirkanmaa Tampere Finland; ^5^ University Consortium of Seinäjoki Seinäjoki Finland; ^6^ Faculty of Social Sciences University of Tampere Tampere Finland; ^7^ New Children's Hospital Helsinki University Hospital Helsinki Finland

**Keywords:** borderline result, coeliac disease, transglutaminase autoantibody

## Abstract

**Background:**

Due to the expanding screening of coeliac disease (CeD), low positive and borderline negative serum transglutaminase 2 antibody (TGA) values are causing increasing confusion in clinical practice.

**Objectives:**

To investigate the significance of these findings in a well‐defined patient cohort.

**Methods:**

Altogether 311 IgA‐competent adults, with clinical suspicion or family history of CeD, underwent duodenal sampling and testing for TGA (ImmunoCAP EliA, cut‐off 7.0 U/mL) and endomysial antibodies (EmA). TGA values 7.0–14.0 U/mL were defined as low positive and 3.0–6.9 U/mL as borderline negative. Besides conventional histology, small bowel mucosal TGA‐targeted IgA deposits and γδ+ intraepithelial lymphocytes (IELs) were determined as CeD‐specific markers.

**Results:**

Twenty‐eight (9%) individuals had low positive TGA, and 22 (79%) were also positive for EmA. Among those with low positive TGA, all EmA positive and 50% of the EmA negative subjects were diagnosed with CeD. Thirty‐nine individuals (13%) had borderline negative TGA, and 36% were positive for EmA. Of these, 79% of EmA positive and 12% of EmA negative subjects were diagnosed with CeD. Additionally, 29% of the subjects with borderline negative TGA and no diagnosis exhibited signs of incipient CeD, including positive IgA deposits, increased density of γδ+ IELs and presence of human leukocyte antigen DQ2/DQ8. All subjects with TGA ≥ 3.2× upper limit of normal (22.4 U/mL) received a CeD diagnosis.

**Conclusion:**

Low positive and borderline negative TGA frequently implies a CeD diagnosis, particularly in EmA positive individuals, or at least may be an indicator of an early stage of the disease.

## Graphical abstract

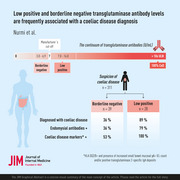

## Introduction

Coeliac disease (CeD) is a gluten‐driven enteropathy, characterized by small bowel mucosal villous atrophy and crypt hyperplasia, disease‐specific autoantibodies against tissue transglutaminase 2 (TGA) and endomysium (EmA) and a diverse clinical presentation [1]. The modern serological tests, especially those measuring TGA and EmA, have in recent decades significantly improved the efficiency of CeD diagnostics [2]. Extensive research has demonstrated a strong correlation between high positive TGA levels and the severity of the CeD‐related histological damage [3, 4, 5, 6]. This has paved the way for the establishment of the no‐biopsy criteria for CeD, initially set for children in Europe and, in some countries, for example, in Finland, also extended to adults [7, 8, 9].

However, there remain unanswered questions, particularly regarding the interpretation of low positive or borderline negative autoantibody results [10]. Individuals with low positive TGA levels (only one to two times the upper limit of normal (ULN)) may not necessarily have the diagnostic enteropathy [11, 12, 13]. Conversely, some cases with borderline negative TGA levels, with or without EmA positivity, may exhibit signs of enteropathy or early stage CeD [10, 14, 15, 16, 17]. Low or negative serology may be caused by the use of immunosuppressant drugs, gluten avoidance or IgA deficiency [18], but often no specific cause is identified. Interpretation of the results is further complicated by a lack of standardization and the variable performance of TGA tests [19]. Overall, more data are needed on these clinically challenging scenarios.

We aimed to investigate the histological and serological findings and clinical significance of low positive and borderline negative TGA values in a well‐defined cohort of adults suspected of having CeD.

## Methods

### Patients and study design

The study was conducted in the Celiac Disease Research Center, Tampere University and at Tampere University Hospital. The cohort consisted of adults with clinical suspicion of CeD or family history of CeD. Those with clinical suspicion were referred to a tertiary centre due to various gastrointestinal and/or extraintestinal symptoms suggestive of CeD. Individuals with familial risk had one or more affected relative(s) and participated in a CeD screening study. The majority (76%) of the participants were first‐degree relatives in the initial study [20]. All participants underwent the collection of research samples used for serological and genetic testing, as well as oesophagogastroduodenoscopy (EGD) with systematic duodenal sampling. Exclusion criteria included age under 18 years, previous diagnosis of CeD, restricted dietary gluten consumption and IgA deficiency (below 0.05 g/L).

The study protocol and patient enrolment were approved by the Regional Ethics Committee of Pirkanmaa Hospital District. The protocol conforms to the ethical guidelines of the Declaration of Helsinki. All study participants were adequately informed and gave written consent to the use of their data and samples for research purposes.

### Clinical data

Demographic and clinical data were collected from all participants. Clinical presentations leading to suspicion of CeD were further categorized as gastrointestinal (e.g., diarrhoea, loose stool, abdominal pain, flatulence and dyspepsia), malabsorption (anaemia and weight loss) and extraintestinal (e.g., bullous rash, arthralgia, osteoporosis, unexplained infertility and neurological manifestations). Participants could be included only in one symptom group based on the most prominent symptoms according to clinical evaluation. Individuals investigated for CeD due to family history were defined as ‘screen‐detected’.

### Serology and CeD‐associated genetics

The commercial ImmunoCAP (Phadia, Freiburg, Germany) assay was used to test serum IgA class TGA. The cut‐off for seropositivity was 7.0 U/mL according to the manufacturer's instructions. The participants were further classified into five subgroups according to their TGA result as follows: low negative (<3.0 U/mL), borderline negative (3.0–6.9 U/mL), low positive (7.0–14.0 U/mL corresponding to 1–2× ULN), intermediate positive (14.1–70.0 U/mL corresponding to >2–10× ULN) and high positive TGA (≥70.1 U/mL corresponding to >10× ULN). Serum IgA class EmA was determined from the same blood sample by indirect immunofluorescence using human umbilical cord tissue as antigen. EmA titres 1: ≥5 were considered positive. The CeD‐associated human leukocyte antigen (HLA) genotypes were tested from whole blood samples, as described elsewhere [8, 21].

### Histology and CeD diagnosis

The EGDs were conducted either at Tampere University Hospital or at some other local primary care unit with experience in CeD diagnostics, using standard protocols and equipment. A minimum of four representative duodenal biopsies was taken during EGD. Bulbus biopsies were not taken. Only correctly orientated mucosal sections were accepted for histopathological analysis [22]. CeD diagnosis was based on the demonstration of mucosal damage equivalent to Marsh 2–3 lesions. A subgroup of individuals with mild enteropathy (Marsh 1) and HLA DQ2 and/or DQ8 genotype underwent a 1‐year trial on gluten challenge and/or gluten‐free diet, after which the investigations were repeated, and the diagnosis was set on the basis of serological, histological and clinical responses [23, 24, 25, 26]. In these cases, additional special CeD‐specific findings were used [23, 24, 25, 26]. These included quantitative determination of the mucosal villous height–crypt depth ratio (Vh/CrD, <2 equivalent for Marsh 3) [22] as well as measurements of mucosal γδ+ (>4.3 cells/mm) intraepithelial lymphocytes (IELs) and CeD‐specific transglutaminase 2‐targeted IgA deposits from frozen sections [24, 25, 26]. Some participants presented with a bullous rash indicative of dermatitis herpetiformis, a skin manifestation of CeD, in which case the diagnosis was confirmed by detecting granular IgA deposits in a skin biopsy [27].

In addition to established CeD diagnosis, individuals carrying the HLA DQ2 and/or DQ8 haplotype and with increased density of γδ+ IELs and/or positive transglutaminase 2‐targeted IgA deposits in the duodenal mucosa were considered to have positive CeD‐specific markers, irrespective of possible CeD diagnosis.

### Statistics

Descriptive data were presented either as numbers of cases with percentages or as medians with quartiles. Categorical variables were analysed using chi‐square test or Fisher's exact test and continuous variables using Kruskal–Wallis test. A *p* value <0.05 was considered significant. SPSS Statistics version 29 (IBM, Armonk, NY, USA) was used to perform the statistical analyses.

## Results

Overall, 311 adults were included, 221 with clinical suspicion and 90 with family risk for CeD. Altogether 192 had positive TGA (≥7.0 U/mL), including 28 with 1–2× ULN, 95 with >2–10× ULN and 69 with >10× ULN. Eighty subjects had a low negative and 39 a borderline negative TGA (Table 1). There was a significant age difference between the groups, individuals with low negative TGA being the youngest and those with borderline negative TGA the oldest. A linear trend between age and TGA levels was not detected. TGA negative subjects had more gastrointestinal and extraintestinal symptoms than did the TGA positive cases, who were more often screen‐detected and relatives of CeD patients (Table 1).

**Table 1:** Characteristics of 311 subjects examined for CeD and categorized into five different levels of TGA^a^.

	Negative TGA		Positive TGA			
	Low negative <3.0 U/mL	Borderline negative 3.0–6.9 U/mL	Low positive 7.0–14.0 U/mL	Intermediate positive 14.1–70.0 U/mL	High positive >70.1 U/mL	
	*n* = 80	*n* = 39	*n* = 28	*n* = 95	*n* = 69	*p* value
Females, *n* (%)	57 (71)	23 (59)	14 (50)	69 (73)	47 (68)	0.143
Age, median (quartiles), year	40 (27, 51)	53 (36, 63)	44 (39, 56)	47 (33, 60)	42 (32, 54)	**0.004**
Clinical presentation, *n* (%)						**<0.001**
Gastrointestinal	60 (75)	27 (69)	11 (39)	51 (54)	21 (30)	
Malabsorption^b^	3 (4)	1 (3)	1 (4)	5 (5)	8 (12)	
Extraintestinal	15 (19)	4 (10)	1 (4)	7 (7)	6 (9)	
Screen‐detected	2 (3)	7 (18)	15 (54)	32 (34)	34 (49)	
Family history of CeD, *n* (%)	10 (13)	10 (26)	17 (61)	49 (52)	37 (54)	**<0.001**
HLA DQ2/8 present, *n* (%)	50 (63)	31 (80)	28 (100)	95 (100)	69 (100)	**<0.001**
EmA positive, *n* (%)	3 (4)	14 (36)	22 (79)	86 (91)	69 (100)	**<0.001**
Histology, *n* (%)						**<0.001**
Marsh 0	54 (68)	17 (44)	3 (11)	4 (4)	0	
Marsh 1	22 (28)	12 (31)	5 (18)	14 (15)	3 (4)	
Marsh 2–3	4 (5)	10 (26)	20 (71)	77 (81)	66 (96)	
CeD‐specific markers^c^, *n* (%)	24 (32)	17 (53)	17 (100)	61 (100)	33 (100)	**<0.001**
CeD diagnosis^d^, *n* (%)	4 (5)	14 (36)	25 (89)	91 (96)	69 (100)	**<0.001**

*Note*: Values in bold face indicate statistically significant values.

Abbreviations: CeD, coeliac disease; EmA, endomysial antibodies; HLA, human leukocyte antigen; TGA, transglutaminase 2 antibody.

^a^ ImmunoCAP EliA test, manufacturer's cutoff 7.

^b^ Malabsorption and/or anaemia.

^c^ HLA DQ2/8 positivity and presence of increased small bowel mucosal γδ+ IEL count and/or positive transglutaminase 2‐specific IgA deposits (data from 219 individuals).

^d^ Either diagnostic lesion (Marsh 2–3, *n* = 177), Marsh 1 + HLA DQ2/8 + histological, serological and clinical response on a gluten‐free diet and/or gluten challenge (*n* = 24), or biopsy‐proven dermatitis herpetiformis (*n* = 2).

**Fig. 1:**
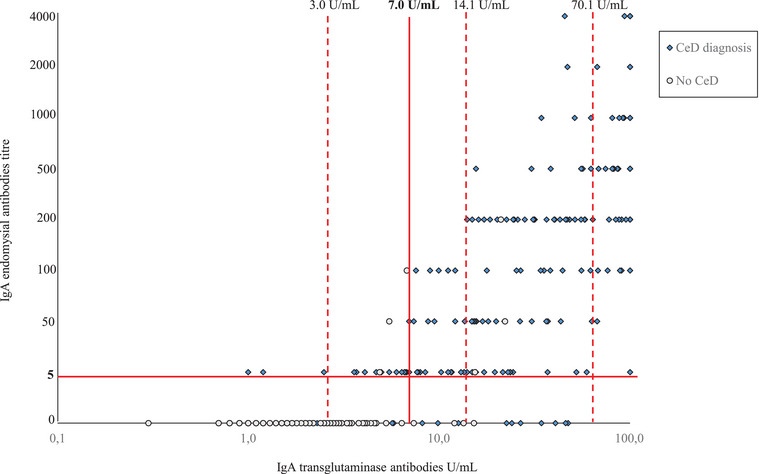
Serum IgA endomysial antibodies and logarithmic representation of the serum IgA transglutaminase antibodies. Red lines indicate concentrations above the upper limit for normal endomysial antibodies and for different upper limits of normal levels of transglutaminase antibodies (borderline negative, low positive 1–2×, intermediate positive >2–10× and high positive >10× ULN).

All participants with positive TGA, regardless of the level, had HLA DQ2/DQ8 haplotype. Individuals with high positive TGA were also all EmA positive, exhibited CeD‐specific histologic markers and were diagnosed with CeD, whereas the corresponding figures in those with intermediate positive (>2–10× ULN) TGA were 91%, 100% and 96% (Table 1). The four subjects with intermediate positive TGA who did not receive a diagnosis all had Marsh 0 histology and TGA values 15.3–22.3 U/mL (2.2–3.2× ULN) (Fig. 1). One was EmA negative and had increased density of γδ+ IELs but no IgA deposits (Fig. S1). The other three were EmA positive but had no frozen biopsies available.

In the low positive TGA group (*n* = 28), 79% had positive EmA, 100% (17/28 measured) had CeD‐specific markers, and 89% (*n* = 25) were diagnosed with CeD (Table 1). Of those with CeD, 20 presented with Marsh 2–3 lesions, whereas five cases had Marsh 1 and received the diagnosis following additional investigations (Fig. S1). All cases with positive EmA and 3/6 of those with negative EmA were diagnosed with CeD. Three EmA negative individuals exhibited Marsh 0 lesions, one of whom had an increased density of γδ+ IELs and IgA deposits, whereas two had no frozen biopsy available (Fig. S1).

Among individuals with negative TGA levels, 80% of subjects with borderline negative TGA and 63% of those with low negative TGA had HLA DQ2/DQ8 haplotype (Table 1). All EmA positive individuals carried HLA DQ2/DQ8.

Of the borderline negative group (*n* = 39), 36% were EmA positive, 53% (32/39 measured) exhibited CeD‐specific markers, and 36% (*n* = 14) had CeD, including 10 with Marsh 2–3 and four with Marsh 1 and additional investigations (Figs. S1 and 2). The diagnosis was set in 79% of EmA positive cases and in 12% of EmA negative cases. Of the subjects who did not receive a diagnosis, CeD‐specific markers were present in six (29%) (Fig. S1).

**Fig. 2:**
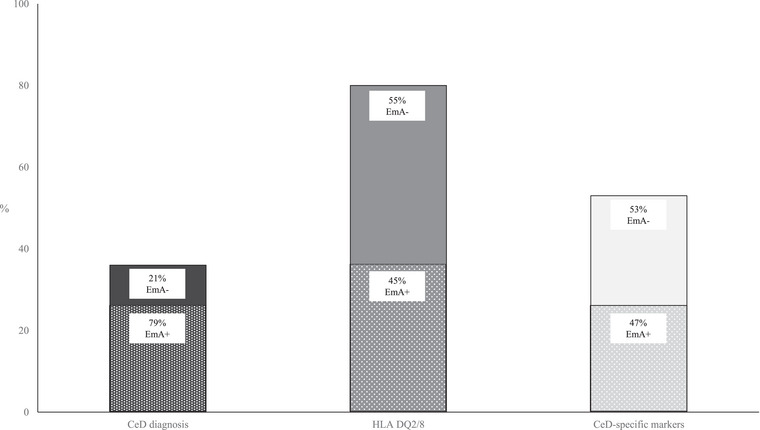
Proportion of coeliac disease (CeD) diagnoses, presence of HLA DQ2/8 and positive CeD‐specific markers among 39 subjects with borderline negative transglutaminase antibodies. Proportion of endomysial antibody positivity (EmA+) and negativity (EmA−) is given in each column. HLA, human leukocyte antigen.

In the low negative TGA group, 4% were EmA positive, and 32% (76/80 measured) exhibited CeD‐specific markers (Table 1). Four individuals (5%) received a CeD diagnosis, including three who were EmA positive (titre 1:5).

Altogether 91% of the 203 participants who received a CeD diagnosis had TGA level ≥7.0 U/mL, whereas 98% had TGA level ≥3.0 U/mL (Fig. 1). The lowest TGA level associated with a 100% CeD diagnosis rate was 3.2× ULN.

## Discussion

We observed that low positive and even borderline negative TGA values frequently led to CeD diagnosis. As screening for CeD becomes more common and TGA is the most widely used first‐line test, these diagnostically challenging scenarios will probably become increasingly common [10, 28, 29, 30]. Overall, CeD patients may present with a wide range of TGA levels and duodenal findings as well as suffer from different symptoms irrespective of the initial clinical suspicion (clinically or screen‐detected) [12, 17]. Both serology and histology should therefore be viewed more as a continuum, and the cut‐off challenges are seen among various TGA tests [31].

Overall, 89% of the patients with low positive TGA were diagnosed with CeD. Previously reported proportions of affected patients among subjects with TGA 1–3 or 1–5× ULN have varied widely, depending on the populations tested and assays used [11, 32, 33]. Several aspects should be considered in individuals with low positive TGA. Instead of indicating false seropositivity, low TGA levels may indicate early stage CeD, in which diagnostic enteropathy has not yet developed [24]. Potential reasons for ‘artificially’ low TGA levels, such as gluten avoidance, the presence of extraintestinal forms of CeD and pitfalls in histological evaluation, should also be considered [18, 34]. In such circumstances, EmA can serve as a useful second‐line test due to its high specificity [35, 36, 37]. All subjects with low positive TGA and positive EmA in this study were diagnosed with CeD. As a challenge, the availability of EmA is limited, it is operator‐dependent [36, 38], and it has shown lower sensitivity than TGA [28, 37].

Of note, as early as in 2012, the European Society for Paediatric Gastroenterology, Hepatology and Nutrition presented that lower than recommended TGA cut‐offs could be used in research studies [30]. With regard to the individuals with borderline negative TGA in our study, 36% were diagnosed with CeD, with the proportion rising to 79% in those who were EmA positive. A few studies have so far explored this issue [14, 15, 16]. In a study by Hill et al., 11 adults had borderline negative TGA levels, seven underwent biopsy, and five were diagnosed with CeD [15], whereas in a Spanish study, CeD was found in two out of six corresponding individuals [14]. A Swedish study reported borderline negative TGA in 104 children, of whom 20 were EmA positive, 18 underwent biopsy and 10 had CeD [16]. These results corroborate our findings and demonstrate that many of these subjects, particularly when EmA positive, have a biopsy‐proven CeD. We also recently found that isolated borderline negative TGA was highly predictive of subsequent CeD development during a follow‐up [10]. Additionally, in a screening study among once seronegative subjects with family risk for CeD, those who later developed the disease had at the first testing approximately 10 years earlier shown higher TGA levels than their unaffected relatives [17].

A major challenge is determining if and when to perform a biopsy in individuals with low positive or borderline negative TGA and negative EmA. There is no international consensus on how to manage these scenarios. Again, it is important first to consider the potential diagnostic pitfalls. HLA determination could help to exclude CeD, although the absence of at‐risk haplotypes is rare among seropositive individuals [28, 37]. One suggested approach is to repeat serological testing after 3–6 months on a gluten‐containing diet in individuals with compatible HLA [28]. If a biopsy is performed, the appropriate number and quality of the samples must be ensured, and seamless communication with an experienced pathologist is essential [1, 28].

The use of special methods to detect early stage histological markers of CeD can also be beneficial [39, 40, 41]. We observed these in more than half of individuals with borderline negative TGA. IgA deposits in particular are highly specific for CeD and could be detectable even in EmA negative patients [40], although testing is currently available only in specialized centres. Of note, endoscopy with duodenal biopsy should be conducted based on clinical decision‐making, regardless of serology results, for instance, in individuals showing signs of malabsorption [28, 37]. Emerging endoscopic, histological and immunological tools are in the near future expected to improve early diagnostics of CeD [42, 43, 44, 45].

The main strength of this study was the well‐defined cohort of individuals with suspected CeD, all of whom underwent serological testing and EGD with duodenal biopsies. Additionally, sophisticated and validated special diagnostic methods were used for a subgroup of individuals with non‐conclusive histology. As a limitation, data on the possible use of medications that may affect the duodenal mucosa were not available. Furthermore, antibodies against deamidated gliadin peptides were not assessed and bulbus biopsies were not taken, although diagnostic pitfalls are possible in the interpretation of the latter [46, 47]. Finally, all study subjects had a high pretest probability for CeD, which limits the generalizability of the findings. Our study described real‐life situation, and it may not be completely comparable with the study populations used in original cut‐off studies. In addition, confirmatory studies on paediatric cohorts are warranted.

To conclude, borderline negative TGA often indicates a diagnosis of CeD or may reflect early stage disease development. Testing for EmA, when available, appears to be particularly useful among subjects with low positive or borderline negative TGA. As such, CeD serology, as well as histology, should be viewed more as a continuum, with greater emphasis placed on the diagnostic findings overall. It is important that physicians encountering CeD patients recognize these concerns and are familiar with the TGA test used in their practice. These issues should be more thoroughly addressed in future diagnostic guidelines, and there is a clear need for improved diagnostic tools.

## Author contributions

**Rakel Nurmi**: Conceptualization; methodology; formal analysis; writing—original draft; visualization. **Celina Turunen Beteta**: Formal analysis; writing—original draft. **Kalle Kurppa**: Conceptualization; writing—review and editing. **Heini Huhtala**: Formal analysis; writing—review and editing. **Katri Lindfors**: Writing—review and editing. **Laura Kivelä**: Conceptualization; writing—review and editing. **Katri Kaukinen**: Conceptualization; methodology, writing—original draft; visualization; investigation. **Saana Paavola**: Conceptualization; methodology; formal analysis; writing—original draft; visualization. All authors approved the final version of the manuscript for publication.

## Conflict of interest statement

Rakel Nurmi has received personal lecture fees from the Finnish Coeliac Society. Celina Turunen Beteta has received a travel grant from the Finnish Coeliac Society. Kalle Kurppa has received personal lecture fees from the Finnish Coeliac Society and Columbia University and consultancy fees from Takeda and Sanofi. Laura Kivelä has received personal lecture fees from the Finnish Coeliac Society and a travel grant from the International Society for the Study of Coeliac Disease and Takeda. Saana Paavola has received a personal lecture fee from the Finnish Coeliac Society and a travel grant from Dr. Falk Pharma. The other authors report no conflicts of interest.

## Funding information

Academy of Finland, Finnish Cultural Foundation, Finnish Medical Foundation (7850), Paulo Foundation, Research Council of Finland (361421), Sigrid Jusélius Foundation and State Research Financing of the Expert Responsibility Area of Tampere University Hospital.

## Supporting information



Supporting Information

## Data Availability

The data that support the findings of this study are not publicly available due to privacy and ethical restrictions.
