# Supplementary material for: Low positive and borderline negative transglutaminase antibody levels are frequently associated with a coeliac disease diagnosis
Source: J Intern Med. 2025 Sep 25;298(6):617–25. doi: 10.1111/joim.70025 (PMC12617477; doi:10.1111/joim.70025)
Supplement: Supplementary file 1 — Supporting Information [file JOIM-298-617-s001.pdf]

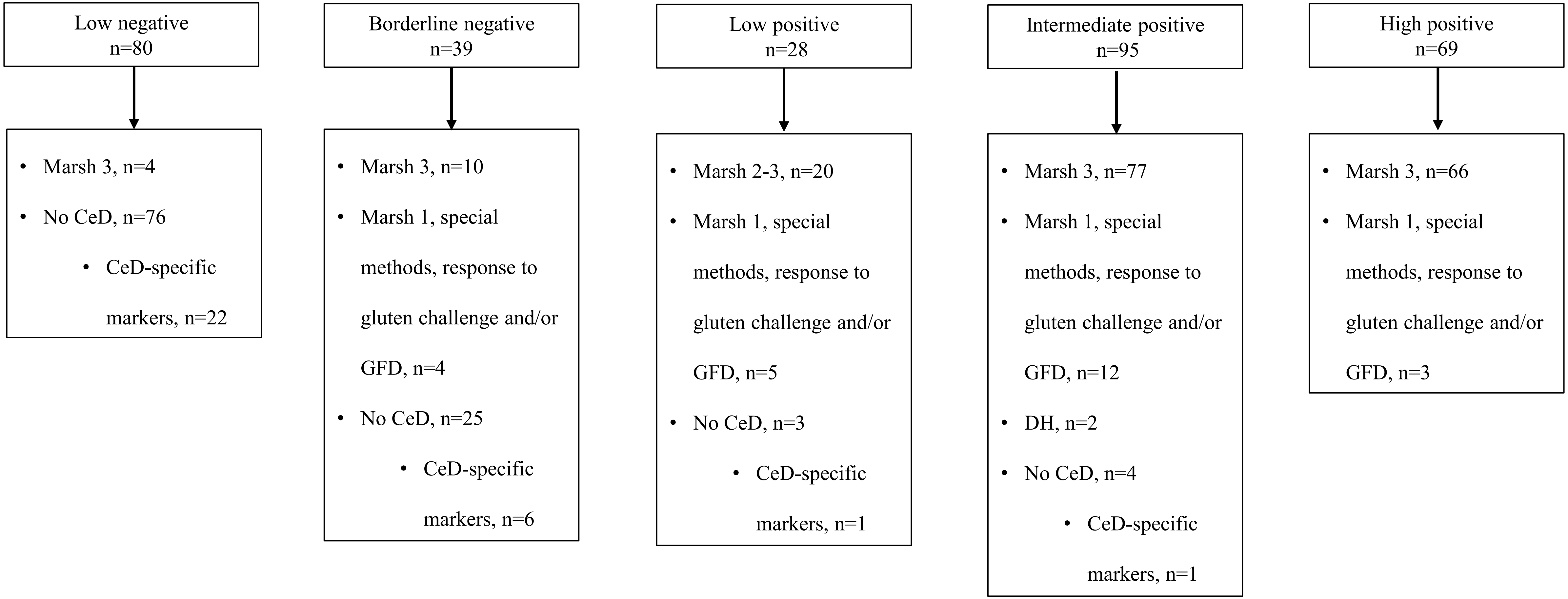
Low negative n=80
Marsh 3, n=4
No CeD, n=76
CeD-specific markers, n=22
Borderline negative n=39
Marsh 3, n=10
Marsh 1, special methods, response to gluten challenge and/or GFD, n=4
No CeD, n=25
CeD-specific markers, n=6
Low positive n=28
Marsh 2-3, n=20
Marsh 1, special methods, response to gluten challenge and/or GFD, n=5
No CeD, n=3
CeD-specific markers, n=1
Intermediate positive n=95
Marsh 3, n=77
Marsh 1, special methods, response to gluten challenge and/or GFD, n=12
DH, n=2
No CeD, n=4
CeD-specific markers, n=1
High positive n=69
Marsh 3, n=66
Marsh 1, special methods, response to gluten challenge and/or GFD, n=3
